# Relativistic analysis of the Michelson-Gale experimental result

**DOI:** 10.1038/s41598-024-60515-7

**Published:** 2024-04-30

**Authors:** Yang-Ho Choi

**Affiliations:** Department of Electrical and Electronic Engineering, Kangwon National University, Chunchon, Kangwon-do 200-701 South Korea

**Keywords:** Michelson-Gale experiment, Coordinate transformation, Standard synchronization, Speed of light, Sagnac effect, Optical physics, Space physics

## Abstract

The result of the Michelson-Gale experiment, which shows fringe shifts by the interference between two light beams traversing a rectangular loop in opposite directions, has been nonrelativistically analyzed based on the Galilean transformation. We relativistically analyze it via the transformation under the constant light speed (TCL) and via the framework of Mansouri and Sexl (MS). The TCL provides a coordinate transformation between the isotropic frame and a rotating frame, in which the two-way speed of light is a constant *c* irrespective of direction on the surface that has the same radius of rotation. When using TCL, we assume that the Solar System is isotropic so that the one-way speed of light is *c* in it. On the contrary, considering its movement, the analysis is carried out without the assumption of isotropy based on the MS framework. The analysis results via the TCL and via the MS framework correspond to each other and are in agreement with the result of the experiment. It is shown that the difference between the travel times of the counter-propagating light beams, which results in the fringe shift, takes place due to the two factors, the anisotropy of the one-way speed of light in inertial frames and the different rotation radii at different latitudes on the Earth surface.

## Introduction

Michelson had shown a great passion to search for the luminiferous ether. He continued his efforts in the Michelson-Gale (MG) experiment^1^, more than 35 years after the null result in the famous Michelson-Morley (MM) experiment^2^, and at last had observed fringe shifts. The MG experiment employed a large rectangular loop with a perimeter of about 1.9 km that two light beams traverse in opposite directions. The fringe shift is due to the difference between the travel times of the counter-propagating light beams that travel the same distance. Though nearly 100 years have passed since then, very few relativistic analyses on the experiment result are found, which may indicate the difficulty that the special and general relativity suffers in consistently handling circular motions^3–5^. It is stated in ref.^6^ that “an imposing list of more than a thousand books and papers on the subject of the velocity of light makes no mention of this experiment.” In ref.^6^, the travel times of the light beams were nonrelativistically analyzed, under the assumption that the speed of light is constant regardless of direction in the Solar System. In ref.^7^, mentioning that the hypothesis of a dragging of the ether is not valid as an explanation about the null result, the MG experiment is invoked.

Circular motions can be consistently dealt with by the transformation under the constant light speed (TCL)^8^, which provides a relativistic coordinate transformation between a uniformly rotating frame $\tilde{S}^{\prime}$ and the isotropic frame $S$. The speed of light is a constant $c$ in the isotropic frame $S$. The two-way speed of light in $\tilde{S}^{\prime}$ is $c$ on the surface that has the same rotation radius. Circular motion can be considered locally and momentarily inertial. Accordingly, a coordinate transformation between *S* and an inertial frame, which is termed the inertial transformation, can be derived from the TCL, which shows that it is consistent with the transformation between inertial frames. When the standard synchronization is employed in the inertial frame the inertial transformation becomes identical to the Lorentz transformation.

The Mansouri-Sexl (MS) framework^9^, which presupposes a privileged isotropic frame, can allow us to generally deal with motions of arbitrary direction. Under the MS framework, circular motions can also be relativistically approached^5,8^. We analyze the travel time difference in the MG experiment via TCL and via the MS framework. In the analysis based on TCL, the Solar System is assumed to be isotropic so that it is regarded as $S$ and then the Earth can be represented as $\tilde{S}^{\prime}$. As a matter of fact, it moves in our galaxy, Milky Way, and its frame would not be isotropic. Without the assumption of isotropy, the experimental result can be investigated using the MS framework. Introducing the standard synchronization of clocks such that the speed of light appears to be isotropic in the Earth and the Solar System, we make the analysis under the unique isotropic frame. These analysis results correspond and are in agreement with the result of the experiment. It has been believed that the one-way speed of light is constant in inertial frames. However, the anisotropy of the speed of light in inertial frames has already been observed empirically in the experiments of the generalized Sagnac effect^10–14^. The fringe shift in the MG experiment is shown to take place due to the anisotropy of the one-way speed of light in inertial frames and the difference in the rotation radii of the two segments, laid at different lines of latitude on the Earth surface, of the rectangular loop.

## Relativistic coordinate transformations

The MG experimental result is relativistically analyzed under the MS framework and under the TCL. Presupposing a preferred frame *S*, the spacetime of which is isotropic so that the speed of light is $c$ in any direction, the MS framework has been derived from fundamental kinematics^9^ and the TCL has been developed based on the Lorentz transformation^8^. In this section, we introduce them.

### In MS framework

We represent spacetime coordinate vectors in a complex Euclidean space where time is expressed as an imaginary number. The coordinate vector of the preferred frame * S* is denoted as ${\mathbf{p}} = [\tau ,\;x,\;y,\;z]^{T}$ where $T$ stands for the transpose and $\tau = ict$ represents imaginary time. An inertial frame $S_{k}$ is in motion at a constant velocity ${\mathbf{v}}_{k}$ relative to *S* and its coordinate vector is designated as ${\mathbf{p}}_{k} = [\tau_{k} ,x_{k} ,\;y_{k} ,\;z_{k} ]^{T}$. The symbol ${\varvec{\beta}}_{k}$ is used to indicate the normalized velocity of ${\mathbf{v}}_{k}$ with respect to $c$, i.e. ${\varvec{\beta}}_{k} = {\mathbf{v}}_{k} /c$. For a vector ${\mathbf{q}}$, we denote its normalized vector by ${\hat{\mathbf{q}}}$ and its magnitude by $q$. For example, $\hat{\user2{\beta }}_{k} = {\varvec{\beta}}_{k} /|{\varvec{\beta}}_{k} |$ and $\beta_{k} = \;|{\varvec{\beta}}_{k} |$ where $\;| \cdot |$ designates the Euclidean norm.

The MS formulation includes three coefficients that have to be determined, allowing for the application of various synchronizations. We introduce the standard synchronization into $S_{k}$ and the standard-synchronized frame is denoted as $S_{k \cdot }$. The coefficients are set according to the special theory of relativity. Then the differential coordinate vector of *S* is transformed into $S_{k \cdot }$ as

1 $$d{\mathbf{p}}_{k} = {\mathbf{T}}_{L} ({\varvec{\beta}}_{k} )d{\mathbf{p}},$$

where ${\mathbf{T}}_{L} ({\varvec{\beta}}_{k} )$ is the Lorentz transformation matrix,

2 $${\mathbf{T}}_{L} ({\varvec{\beta}}_{k} ) = \left[ {\begin{array}{*{20}c} {\gamma_{k} } & { - i\gamma_{k} {\varvec{\beta}}_{k}^{T} } \\ {i\gamma_{k} {\varvec{\beta}}_{k} } & {(\gamma_{k} - 1)\hat{\user2{\beta }}_{k} \hat{\user2{\beta }}_{k}^{T} + {\mathbf{I}}} \\ \end{array} } \right],$$

with,

3 $$\gamma_{k} = (1 - |{\varvec{\beta}}_{k} |^{2} )^{ - 1/2} ,$$

and ${\mathbf{I}}$ an identity matrix. Since $d{\mathbf{p}} = {\mathbf{T}}_{L}^{ - 1} ({\varvec{\beta}}_{i} )d{\mathbf{p}}_{i}$, the transformation from one inertial frame $S_{i \cdot }$ to another $S_{j \cdot }$ is expressed as^5,15^.

4 $$d{\mathbf{p}}_{j} = {\mathbf{T}}_{L} ({\varvec{\beta}}_{j} ,\;{\varvec{\beta}}_{i} )d{\mathbf{p}}_{i} ,$$

where,

5 $${\mathbf{T}}_{L} ({\varvec{\beta}}_{j} ,\;{\varvec{\beta}}_{i} ) = {\mathbf{T}}_{L} ({\varvec{\beta}}_{j} ){\mathbf{T}}_{L}^{ - 1} ({\varvec{\beta}}_{i} ).$$

It is obvious that ${\mathbf{T}}_{L}^{ - 1} ({\varvec{\beta}}_{k} ) = {\mathbf{T}}_{L}^{T} ({\varvec{\beta}}_{k} )$, which leads to ${\mathbf{T}}_{L}^{ - 1} ({\varvec{\beta}}_{j} ,\;{\varvec{\beta}}_{i} )\, = {\mathbf{T}}_{L}^{T} ({\varvec{\beta}}_{j} ,\;{\varvec{\beta}}_{i} )$.

Proper time (PT) is independent of synchronization schemes and can be obtained in any inertial frame if relative velocity is known. We use a subscript ‘$\circ$’ in PT to distinguish it from the adjusted time (AT) through the synchronization of clocks. The PT interval is measured at the same place. From (1) and (4), the PT interval of an observer $O_{j}$ who is at rest in $S_{j}$ is expressed as $d\tau_{{j^{^\circ } }} = d\tau_{i} /\gamma_{ji} = d\tau /\gamma_{j}$, which is valid even if $i$ and $j$ are interchanged.

### In TCL

An observer $\tilde{O^{\prime}}$ is located at a radius $r^{\prime}$ in a primed rotating frame $\tilde{S}^{\prime}$, the coordinate vector of which is represented as ${\mathbf{\tilde{p}^{\prime}}}\, = [t^{\prime},\;r^{\prime},\;\tilde{\varphi^{\prime}},z^{\prime}]^{{T}}$ in the cylindrical coordinate system where $\tilde{\varphi^{\prime}}$ indicates an azimuth angle. The observer is rotating at an angular velocity $\omega$ in the isotropic frame *S*, the coordinate vector of which is denoted by ${\mathbf{p}}\, = [t,\;r,\;\varphi ,z]^{T}$. In TCL, the coordinate transformation between $\tilde{S}^{\prime}$ and *S* is given by,

6 $$t^{\prime}\, = \frac{t}{\gamma },\,r^{\prime} = \gamma {\kern 1pt} r\,,\,\tilde{\varphi^{\prime}} = \varphi - \omega \,t,\,z^{\prime} = z.$$

where $\gamma = (1 - \beta^{2} )^{ - 1/2}$ with $\beta = r\omega /c$. The elapsed time and the radius in the primed are different from those in the unprimed. As a result, the angular velocity $\omega^{\prime}$ as seen in the primed becomes different from $\omega$. It is convenient to introduce the primed inertial frame $S^{\prime}$ corresponding to *S* and the unprimed rotating frame $\tilde{S}$ corresponding to $\tilde{S}^{\prime}$. The coordinate transformations between *S* and $\tilde{S}$ in the unprimed and between $S^{\prime}$ and $\tilde{S}^{\prime}$ in the primed are nonrelativistic Galilean. The azimuth angle $\varphi^{\prime}$ in *S′* is the same as $\varphi$ in *S*^8^. The $\tilde{S}$ rotates at the angular velocity $\omega$ in *S* while the $\tilde{S}^{\prime}$ does at the angular velocity $\omega^{\prime}$ in $S^{\prime}$, where $\omega^{\prime}$ and $\omega$ are related by $\omega^{\prime} = \gamma {\kern 1pt} \omega$^8^. It should be noted that $r^{\prime}$ is the radius seen by the observer moving with the tangential speed of $r^{\prime}\omega^{\prime}$ in $S^{\prime}$.

The two-way speed of light is the constant $c$ in TCL^8^. In other words, when $r^{\prime}$ is fixed, the two-way speed is constant regardless of direction in TCL, which is consistent with the result of the MM experiment.

## Analysis of the MG experiment result

**Figure 1:**
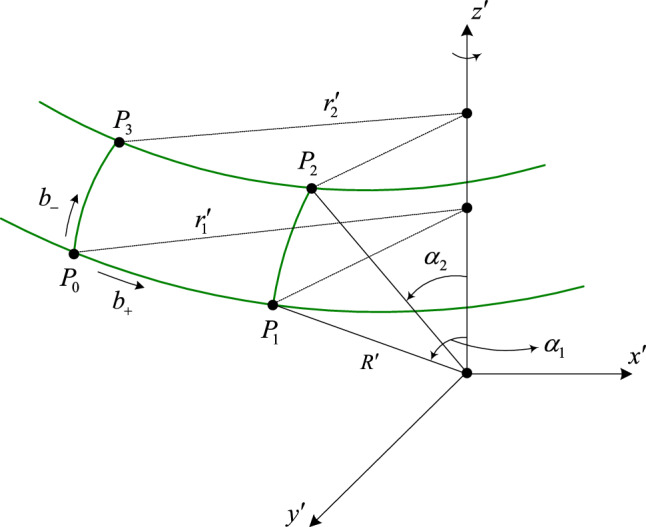
Closed loop in the MG experiment.

We investigate the result of the MG experiment with the transformation (6) in Subsection "With TCL", assuming that the Solar System is isotropic, and based on the MS framework without the assumption in Subsection "Based on the MS framework". Michelson had speculated about an interferometer to measure the Sagnac effect^16^ by the rotation of the Earth and, together with Gale and Pearson in 1925, carried out the experiment using a large rectangular loop. Figure 1 illustrates the closed loop for the MG experiment laid on the surface of the Earth. The angular velocity of the Earth is $$\omega$$ as seen in the Solar System. The light source and detector are located at the same place $$P_{0}$$. Two light beams emitted from the source at the same time travel along the closed loop in opposite directions. We denote by $$b_{ + }$$ and $$b_{ - }$$ the light beams leaving the source in the horizontal and vertical directions respectively. It is assumed that $$R^{\prime}_{1} = R^{\prime}_{2}$$$$( = R^{\prime})$$ where $$R^{\prime}_{m}$$ is the radius of the Earth seen by an observer at the location $$P_{m}$$, $$m = 1,\;2$$. The polar angle is $$\alpha_{m}$$ at $$P_{m}$$ and the radius of rotation is written as,

7 $$r^{\prime}_{m} = R^{\prime}\sin \alpha_{m} .$$

The segments $P_{0} P_{3}$ and $P_{1} P_{2}$ have the same length of $l^{\prime}_{h}$ and $\alpha_{1}$ and $\alpha_{2}$ are related by $\alpha_{1} = \alpha_{2} + \Delta \alpha$ where $\Delta \alpha = l^{\prime}_{h} /R^{\prime}$. The azimuthal angles that subtend the arcs $P_{0} P_{1}$ and $P_{2} P_{3}$ are equal to $\Delta \tilde{\varphi^{\prime}}$. Their lengths are given by.

8 $$l^{\prime}_{wm} = r^{\prime}_{m} \Delta \tilde{\varphi^{\prime}},\,m = 1,\;2.$$

When the light beams $b_{ + }$ and $b_{ - }$ return to the detector, their travel times are different, which brings about a fringe shift. The times that are taken for $b_{ + }$ and $b_{ - }$ to transverse the segments $P_{0} P_{3}$ and $P_{1} P_{2}$, by symmetry between them, are equal and thus the travel time difference results from the others. For convenience, we use $L_{1}$ and $L_{2}$ to represent the segments $P_{0} P_{1}$ and $P_{3} P_{2}$, respectively.

### With TCL

The Solar System is assumed to be isotropic. Our Earth and Solar System correspond to $\tilde{S}^{\prime}$ and *S*, respectively. The angular velocity of $\tilde{S}$ is $\omega$ in *S* while that of $\tilde{S}^{\prime}$ is $\omega^{\prime}( = \gamma {\kern 1pt} \omega )$ in $S^{\prime}$. If we know the speeds in $\tilde{S}^{\prime}$ of $b_{ \pm }$ their travel times can be calculated. The speed of light is known in *S*. Using the speed of light in *S*, we can obtain the speeds of $b_{ \pm }$ in $\tilde{S}^{\prime}$. If $r^{\prime}$ is fixed, so is $r$ and vice versa. Then the squared line element on the surface of a cylinder of radius $r$ is written in *S* as,

9 $$ds^{2} = - c^{2} dt^{2} + \;\;r^{2} d\varphi^{2} + dz^{2} .$$

Substituting (6) into (9) gives,

10 $$ds^{2} = - c^{2} (\gamma {\kern 1pt} dt^{\prime})^{2} + \;r^{{\prime}{2}} (d\tilde{\varphi^{\prime}} + \omega^{\prime}dt^{\prime})^{2} /\gamma^{2} + dz^{{\prime}{2}} .$$

The differential interval is independent of $dr^{\prime}$ since $r^{\prime}$ is fixed. Noting $r^{\prime}\omega^{\prime} = c\gamma^{2} \beta$, (10) is rewritten as,

11 $$ds^{2} = - (cdt^{\prime})^{2} + \;2\beta \,r^{\prime}d\tilde{\varphi^{\prime}}(cdt^{\prime}) + r^{{\prime}{2}} d\tilde{\varphi^{\prime}}^{2} /\gamma^{2} + dz^{{\prime}{2}} .$$

For light signals, $ds$ reduces to zero, which leads to,

12 $$cdt^{\prime} = \beta \,r^{\prime}d\tilde{\varphi^{\prime}} + dl^{\prime},$$

where $dl^{\prime} = (r^{{\prime}{2}} d\tilde{\varphi }^{{\prime}{2}} + \;dz^{{\prime}{2}} )^{1/2}$. It can be easily shown from (12) that the two-way speed is the constant $c$ irrespective of direction. Suppose that a light beam takes a round trip along a differential $dl^{\prime}$. The sign of $d\tilde{\varphi^{\prime}}$ at one path in the round trip is reversed at the other. The round trip time is thus $dt^{\prime}_{ \updownarrow } = 2dl^{\prime}/c$, and the two-way speed of light becomes $c^{\prime}_{ \updownarrow } = c$. When a light beam traverses $L_{1}$ or $L_{2}$, $dz^{\prime}$ is zero and $dl^{\prime} = r^{\prime}|d\tilde{\varphi^{\prime}}|$. The speeds of the co-rotating and counter-rotating light beams, which are denoted by $c^{\prime}_{ + }$ and $c^{\prime}_{ - }$ respectively, are given by,

13 $$c^{\prime}_{ \pm } = \frac{{dl^{\prime}}}{{dt^{\prime}}} = \frac{c}{1 \pm \beta }.$$

According to the second equation of (6), $r^{\prime}_{m}$ and $r_{m}$, $m = 1,\;2$, are related by $r^{\prime}_{m} = \gamma_{m} r_{m}$ with $\gamma_{m} = (1 - \beta_{m}^{2} )^{ - 1/2}$ where $\beta_{m} = r_{m} \omega /c$. From (13), the elapsed times of $b_{ \pm }$ during the travel to the segment $L_{1}$ are calculated, respectively as,

14 $${\kern 1pt} t^{\prime}_{1 \pm } = \frac{{(1 \pm \;\beta_{1} \,)\,l^{\prime}_{w1} }}{c},$$

and in the case of the travel to $L_{2}$,

15 $$t^{\prime\prime}_{2 \pm } = \frac{{(1 \mp \beta_{2} \,)\,l^{\prime}_{w2} }}{c}.$$

One can also confirm from (14) and (15) that the two-way speed of light is $c$. For example, $c^{\prime}_{1 \updownarrow } = 2\,l^{\prime}_{w1} /({\kern 1pt} t^{\prime}_{1 + } + t^{\prime}_{1 - } ) = c$ where $c^{\prime}_{1 \updownarrow }$ is the two-way speed at $L_{1}$. The elapsed times $t^{\prime\prime}_{2 \pm }$ are measured at $L_{2}$. What we try to attain is the time difference at the detector, which is located at $L_{1}$. Therefore the $t^{\prime\prime}_{2 \pm }$ should be converted into the times by the clock of the detector, which are written as

16 $${\kern 1pt} t^{\prime}_{2 \pm } = \xi_{21} t^{\prime\prime}_{2 \pm } ,$$

where $\xi_{21} = \gamma_{2} /\gamma_{1}$. The time intervals $t^{\prime\prime}_{2 \pm }$ at $L_{2}$ are observed as ${\kern 1pt} \gamma_{2} t^{\prime\prime}_{2 \pm }$ in *S*, which correspond to ${\kern 1pt} t^{\prime}_{2 \pm }$ when seen by the clock of the detector. The difference between the travel times of $b_{ \pm }$ is expressed as,

17 $$\Delta {\kern 1pt} t^{\prime}_{d} = {\kern 1pt} \;t^{\prime}_{ + } - t^{\prime}_{ - } = \frac{{2(\beta_{1} {\kern 1pt} l^{\prime}_{w1} - \xi_{21} \beta_{2} {\kern 1pt} l^{\prime}_{w2} )}}{c},$$

where $$t^{\prime}_{ \pm } = t^{\prime}_{1 \pm } + \,{\kern 1pt} t^{\prime}_{2 \pm }$$.

The tangential speed at the equator is less than 500 m/s, and $\beta_{1}^{{}} ,\;\beta_{2}^{{}} < < 1$. The fringe shift $N$ is given, to a first-order approximation by,

18 $$N = \frac{{4l^{\prime}_{w1} l^{\prime}_{h} \omega^{\prime}\cos \alpha_{1} }}{\lambda c},$$

where $\lambda$ is the wavelength of light. For derivation of (18), see the Supplementary Information. The quantity $l^{\prime}_{w1} l^{\prime}_{h}$ corresponds to the area of the rectangular loop. Equation (18) agrees with the result of the MG experiment^1^.

### Based on the MS framework

In reality, our Solar System moves in the Milky Way and it would be different from the isotropic frame $S$. Though it moves, we can consider that it belongs to an inertial frame during a very short time that the light beams traverse the closed loop. We denote the Solar System by $S_{i \cdot }$, which includes the orbital motion of the Earth. The speed of light is $c$ with respect to AT, $t_{i}$, in $S_{i \cdot }$. The closed loop in Fig. 1 is divided into an infinite number of differential elements. A differential segment $d{\mathbf{l}}_{j}$, which can be located on the segment $L_{1}$ or $L_{2}$, belongs to an inertial frame $S_{j \cdot }$. The direction of $d{\mathbf{l}}_{j}$ is defined such that it is the same as the direction of travel of the light beam $b_{ + }$.

From (4), $|d{\mathbf{p}}_{i} |\; = \;|d{\mathbf{p}}_{j} |$. Since $S_{i \cdot }$ and $S_{j \cdot }$ are standard-synchronized, the time that is taken for a light beam to travel a distance $dl_{j}$ is $dl_{j} /c$ and so $d\tau_{j} = idl_{j}$. When $d{\mathbf{p}}_{j} = [idl_{j} ,\;d{\mathbf{l}}_{j}^{T} ]^{T}$, the corresponding differential vector in $S_{i \cdot }$ is $d{\mathbf{p}}_{i} = [d\tau_{i} ,\;d{\mathbf{l}}_{i}^{T} ]^{T}$. For the light travel, $|d{\mathbf{p}}_{j} |\; = 0$ and thus $d\tau_{i}^{2} + \;|d{\mathbf{l}}_{i} |^{2} = 0$. Equivalently,

19 $$dl_{i}^{{}} = \;|d\tau_{i} |.$$

The differential vector $d{\mathbf{p}}_{i}$ is related to $d{\mathbf{p}}_{j}$ by $d{\mathbf{p}}_{i} = {\mathbf{T}}_{L} ({\varvec{\beta}}_{i} ,\;{\varvec{\beta}}_{j} )d{\mathbf{p}}_{j}$. Equation (19) indicates that $dl_{i}^{{}}$ can be obtained if the first row of ${\mathbf{T}}_{L} ({\varvec{\beta}}_{i} ,\;{\varvec{\beta}}_{j} )$ is known so that $d\tau_{i}$ is found, even though the rest is unknown. The first row of ${\mathbf{T}}_{L} ({\varvec{\beta}}_{i} ,\;{\varvec{\beta}}_{j} )$ is given by ${\mathbf{T}}_{L} ({\varvec{\beta}}_{i} ,\;{\varvec{\beta}}_{j} )_{1r} = \gamma_{ij} [1,\; - i{\varvec{\beta}}_{ij}^{T} ]$ where ${\varvec{\beta}}_{kl}$ is the normalized velocity of $S_{k \cdot }$ relative to $S_{l \cdot }$^5,15^. Then $d\tau_{i}^{{}}$ is calculated as,

20 $$d\tau_{i}^{{}} = i\gamma_{ij} (dl_{j}^{{}} - {\varvec{\beta}}_{ij}^{T} d{\mathbf{l}}_{j}^{{}} ).$$

In the travel of $b_{ + }$($b_{ - }$), $d{\mathbf{l}}_{j}^{{}}$ and ${\varvec{\beta}}_{ij}^{{}}$ are in opposite directions at $L_{1}$($L_{2}$) and in the same direction at $L_{2}$($L_{1}$). Recall $\beta_{m} = r_{m} \omega /c$, $m = 1,\;2$. At $L_{1}$, $\gamma_{ij} = \gamma_{1}$, where $\beta_{ij} = \beta_{1}$, and ${\varvec{\beta}}_{ij}^{T} d{\mathbf{l}}_{j}^{{}} = \mp dl_{j}$ for $b_{ \pm }$ respectively. At $L_{2}$, $\beta_{ij} = \beta_{2}$, $\gamma_{ij} = \gamma_{2}$, and ${\varvec{\beta}}_{ij}^{T} d{\mathbf{l}}_{j}^{{}} = \pm dl_{j}$ for $b_{ \pm }$. The travel distances in $S_{i \cdot }$ of $b_{ \pm }$ at $L_{m}$, $m = 1,\;2$, each are given from (19) and (20) by,

21a $$l_{1 \pm } = (1 \pm \beta_{1} )\,\gamma_{1} l^{\prime}_{w1} ,$$

21b $$l_{2 \pm } = (1 \mp \beta_{2} )\gamma_{2} l^{\prime}_{w2} ,$$

and the travel times are $t_{m \pm } = l_{m \pm } /c$. Then the time difference in *S*_i_ is calculated as,

22 $$\Delta t_{d} = \sum\limits_{m = 1}^{2} {(t_{m + } - \;t_{m - } )} = \frac{{2(\beta_{1} \gamma_{1} l^{\prime}_{w1} - \beta_{2} \gamma_{2} l^{\prime}_{w2} )}}{c}.$$

The time difference at $P_{0}$ is,

23 $$\Delta t^{\prime}_{d} = \frac{{\Delta t_{d} }}{{\gamma_{1} }}.$$

Equation (23) is the same as (17) and is valid regardless of whether the spacetime of *S*_i∙_ is actually isotropic or not.

The time intervals $t_{m \pm }$ in the unprimed correspond to $t^{\prime}_{m \pm } = t_{m \pm } /\gamma_{m}$ in the primed. The speeds of $b_{ \pm }$ at $L_{m}$ are written from $t_{m \pm } = l_{m \pm } /c$ and (21a, 21b) as,

24 $$c^{\prime}_{m \pm } = \frac{{l^{\prime}_{wm} }}{{t^{\prime}_{m \pm } }} = \frac{c}{{1 \mp ( - 1)^{m} \beta_{m} }},\,m = 1,\;2.$$

Equation (24) is consistent with (13). Virtually $L_{1}$ and $L_{2}$ can be considered to belong to certain inertial frames during the very short time of the light travel. As shown in (24), the inertial frames are anisotropic, the speed of light depending on the propagation direction, which has also been observed in the experiments of the generalized Sagnac effect^11–13^. The time difference is caused due to two factors. One is the anisotropy of the light speed at $L_{1}$ and $L_{2}$ each. The other is the difference between the rotation radii of $L_{1}$ and $L_{2}$, which results in different tangential speeds. Although the speed of light is anisotropic, there would be no time difference, as can be seen from (22), if there were no difference in radius, i.e. $r^{\prime}_{1} = r^{\prime}_{2}$. Although the radii are different, no fringe shifts would occur if the speed of light were isotropic in inertial frames.

## Discussion

To find exact physical quantities, we have to use ${\mathbf{T}}_{L} ({\varvec{\beta}}_{j} ,\;{\varvec{\beta}}_{i} )$. However, the absolute velocities ${\varvec{\beta}}_{i}$ and ${\varvec{\beta}}_{j}$ are unknown and we cannot. Disguising the inertial frame $S_{i}$ as isotropic via the standard synchronization and then using ${\mathbf{T}}_{L} ({\varvec{\beta}}_{ji} )$ instead of ${\mathbf{T}}_{L} ({\varvec{\beta}}_{j} ,\;{\varvec{\beta}}_{i} )$, nonetheless, we can exactly obtain some physical quantities such as PTs, Doppler shifts, spatial lengths, and speeds with respect to PT^5^. It is because the first rows of ${\mathbf{T}}_{L} ({\varvec{\beta}}_{j} ,\;{\varvec{\beta}}_{i} )$ and ${\mathbf{T}}_{L} ({\varvec{\beta}}_{ji} )$ are identical^5,15^. One can readily see in the analysis of Subsection "Based on the MS framework" that even if ${\mathbf{T}}_{L} ({\varvec{\beta}}_{ji} )$ is used in place of ${\mathbf{T}}_{L} ({\varvec{\beta}}_{j} ,\;{\varvec{\beta}}_{i} )$ the same time difference as (22) is obtained. A similar disguise via the standard synchronization can be introduced to the TCL as well.

**Figure 2:**
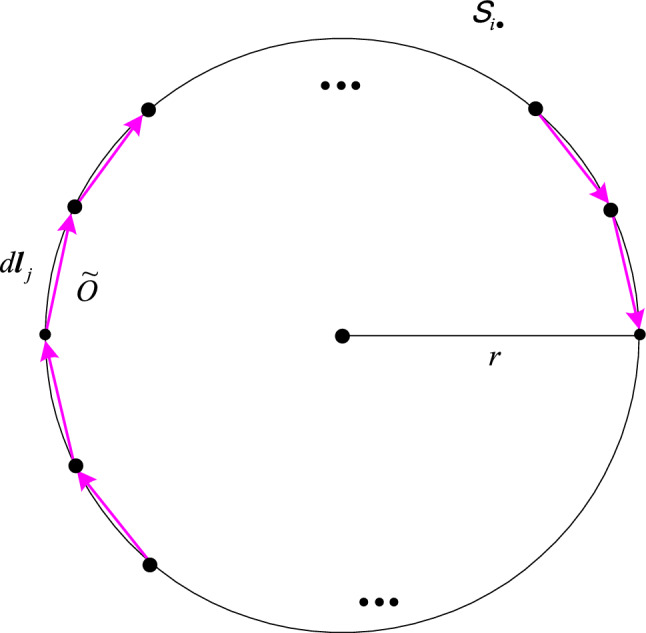
Approximation to a circle with line segments.

An inertial frame $S_{i \cdot }$ that is in motion with a constant velocity of ${\varvec{\beta}}_{i}$ is standard-synchronized. In Fig. 2, a circle of radius $r$ is rotating with an angular velocity $\omega$ in $S_{i \cdot }$. The circle is approximated as $n$ line segments so that circular motion can be treated as rectilinear motion at each segment. As $n$ tends to infinity, the linearized shape becomes a circle. The line segments momentarily belong to inertial frames the speeds of which are all equal to $r\omega$. As seen in $S_{i \cdot }$, an observer $\tilde{O}$ is located at a line segment $d{\mathbf{l}}_{j}$, whose direction varies as the circle rotates. The primed observer corresponding to the unprimed $\tilde{O}$ is $\tilde{O}^{\prime}$, whose coordinate system is also standard-synchronized. In the coordinate transformation associated with $\tilde{O}$ and $\tilde{O}^{\prime}$, as a matter of fact, the observer $\tilde{O}$ represents an observer in $S_{i \cdot }$ who instantaneously meets $\tilde{O}^{\prime}$ as the circle rotates. The $\tilde{O}^{\prime}$ instantaneously moves with the velocity ${\varvec{\beta}}_{ji}$ relative to the observer in $S_{i \cdot }$ represented by $\tilde{O}$. The rotating frame $\tilde{S}^{\prime}$ is formed by the collection of the world lines of these primed rotating observers^8,17^. In other words, the word lines of the primed observers $\tilde{O}^{\prime}_{{j_{k} }}$ corresponding to the unprimed $\tilde{O}_{{j_{k} }}$ located at $d{\mathbf{l}}_{{j_{k} }}$, $k = 1,\;2,\; \cdots$, in $S_{i \cdot }$ constitute $\tilde{S}^{\prime}$.

Suppose that momentarily $\tilde{O}^{\prime}$ belongs to an inertial frame $S_{j \cdot }$, the velocity of which is ${\varvec{\beta}}_{ji}$ in $S_{i \cdot }$. Then the transformation matrix between $S_{j \cdot }$ and $S_{i \cdot }$ is ${\textbf{T}}_{L} ({\varvec{\beta}}_{j} ,\;{\varvec{\beta}}_{i} )$, not ${\mathbf{T}}_{L} ({\varvec{\beta}}_{ji} )$. The transformation (6) has been derived based on the Lorentz transformation for $\tilde{O}$ and $\tilde{O}^{\prime}$. As mentioned above, even if ${\mathbf{T}}_{L} ({\varvec{\beta}}_{ji} )$ is employed we can find exact PTs and exact spatial lengths, which leads us to suggest the transformation between $S_{i \cdot }$ and $\tilde{S}^{\prime}$,

25 $$t^{\prime} = \frac{{t_{ \cdot } }}{\gamma },\,r^{\prime} = \gamma {\kern 1pt} r,\,\tilde{\varphi^{\prime}} = \varphi - \omega \,t_{ \cdot } ,\,z^{\prime} = z.$$

where the symbol $t_{ \cdot }$ is used to explicitly indicate the standard-synchronized time, AT, in $S_{i \cdot }$. The events that occur at the same $t^{\prime}$ in (6) is actually simultaneous whereas the events at the same $t_{ \cdot }$ in (25) is not since $t_{ \cdot }$ is AT. However $t^{\prime}$ is exact since it represents the PT interval.

The analysis of the MG experimental result with (25) is the same as in Subsection "With TCL" except that $t$ is replaced by $t_{ \cdot }$. Here, using (25), we analyze the Sagnac effect. In the experiment of the Sagnac effect, the Earth can be considered to be in linear motion during the traverse of light beams, though it rotates. The inertial frame $S_{i \cdot }$ represents the one for a laboratory. The light detector $\tilde{O^{\prime}}$ is located on a circumference of radius $r$ in $S_{i \cdot }$. At $t_{ \cdot } = t^{\prime} = 0$, two light beams $b_{ + }$ and $b_{ - }$ leave a light source, which is located at the same place as the detector, and traverse the circular paths in the co- and counter-rotating directions respectively.

Since the transformation (25) has the same form as (6), the same equation as (12) is obtained for the former. The angle $\tilde{\varphi^{\prime}}$ is positive in the same direction as the rotation direction of $\tilde{O^{\prime}}$. Integrating (12) with respect to $\tilde{\varphi^{\prime}}$ after the replacement of $dl^{\prime}$ by $r^{\prime}\,|d\tilde{\varphi^{\prime}}|$, we have,

26 $$ct^{\prime}_{ \pm } = \int_{0}^{ \pm 2\pi } {\beta \,r^{\prime}d\tilde{\varphi^{\prime}}} + \int_{0}^{ \pm 2\pi } {r^{\prime}|d\tilde{\varphi^{\prime}}} |\; = (1 \pm \beta )l^{\prime},$$

where $l^{\prime} = 2\pi {\kern 1pt} r^{\prime}$. The travel times of $b_{ \pm }$ are,

27 $$t^{\prime}_{ \pm } = \frac{{(1 \pm \beta ){\kern 1pt} l^{\prime}}}{c}.$$

The time difference is given by,

28 $$\Delta {\kern 1pt} t^{\prime} = \frac{{2\beta {\kern 1pt} l^{\prime}}}{c},$$

which corresponds to the experimental result. The travel distances of $b_{ \pm }$ are $l^{\prime}$ and the speeds of $b_{ \pm }$ with respect to PT are equal to (13).

Using (25), let us make analysis on the travel of the light beams in $S_{i \cdot }$. From (25), $d\varphi = d\tilde{\varphi^{\prime}} + \omega \,dt_{ \cdot }$ and $dt_{ \cdot } = \gamma {\kern 1pt} dt^{\prime}$. While the light beams $b_{ \pm }$ traverse the circular loop, $\tilde{\varphi^{\prime}}$ and $t^{\prime}$ vary from 0 to $\pm 2\pi$ and from 0 to $t^{\prime}_{ \pm }$. Integrating $d\varphi$ yields,

29 $$\varphi_{ \pm } = \pm 2\pi + \gamma {\kern 1pt} \omega \,t^{\prime}_{ \pm } .$$

The travel distances are calculated using (29) and (27) as,

30 $$l_{ \pm } = r|\varphi_{ \pm } |\; = \frac{l}{1 \mp \beta },$$

where $l = 2\pi {\kern 1pt} r$. The speed of light is $c$ with respect to AT and the travel times of $b_{ \pm }$ measured by AT in $S_{i \cdot }$ are given by $t_{ \cdot \pm } = l_{ \pm } /c$, which agrees with (27), i.e. $t_{ \cdot \pm } = \gamma {\kern 1pt} t^{\prime}_{ \pm }$. These analysis results substantiate the transformation (25).

Traditionally the Sagnac effect has been analyzed usually using the Langevin metric [e.g. Refs.^3,4,7,18^]. Since it is the first-order effect of $\beta$ as shown in (28), we can approximately calculate the time difference with the Langevin metric. Neglecting the terms with higher degrees than $\beta$ in (25) yields,

31 $$\tilde{t^{\prime}} = t_{ \cdot } ,\,\tilde{r^{\prime}} = \;{\kern 1pt} r,\,\tilde{\varphi^{\prime}} = \varphi - \omega \,t_{ \cdot } ,\,\tilde{z^{\prime}} = z.$$

where the symbol “tilde” is used to explicitly represent the coordinates of $\tilde{S}^{\prime}$. The Langevin metric is found in accordance with (31). Clearly the transformation (31) is Galilean, which does not recognize the difference between $\tilde{t^{\prime}}$ and $t_{ \cdot }$ and between ${\kern 1pt} \tilde{r^{\prime}}$ and $r$. If the symbol “prime” in the coordinates of $\tilde{S}^{\prime}$ is removed so that for example, $\tilde{r} = \;{\kern 1pt} r$ and if $S_{i \cdot } = S$, (31) becomes the transformation between *S* and $\tilde{S}$, from which the same time difference as (28) is exactly derived with the recognition of the difference between $\tilde{S}$ and $\tilde{S}^{\prime}$ [^8^, p. 184]. Without the recognition of the difference, the computation results using (31) are only valid within the first-order approximation. In the MG paper^1^, the fringe shift, which also results from the first-order effect as in (22), has been calculated based on (31). The frame $\tilde{S}$ is different from $\tilde{S}^{\prime}$. Under $\tilde{S} = \tilde{S}^{\prime}$, the analyses by the Langevin metric or (31) are approximate and nonrelativistic.

Meanwhile, the MM experiment was devised to test the effect of $\beta^{2}$ on the round trip velocity. The Langevin metric, in which the round trip speed of light is anisotropic, fails to explain the MM experiment whereas the TCL of (25) can do. The experiment had been carried out to measure the effect due to the motion of the Earth relative to the Solar System and the two arms of the interferometer, which are very small compared with the radius of the Earth, can be considered to be laid at the same rotation radius. The round trip speed of light is constant in (25) with the radius fixed irrespective of direction. The TCL is consistent with both MM and MG experiments. It is stated in Ref.^18^ that “For uniform rotation in the case of the Sagnac effect one would expect on intuitive grounds that a Galilean rotation (absolute time) might give the correct choice of spacetime coordinate transformation. In consideration, however, of well-known experiences with electromagnetic theory in the realm of uniform translations where the Galilean translation (absolute time) is not an adequate substitute for a Lorentz translation, it is useful to give special attention to the question of selecting the right transformation for uniform rotations.”

## Conclusion

The result of the MG experiment has been analyzed via the TCL and via the MS framework. These analysis results correspond and agree with the experimental result. In the MG experiment, the difference between the travel times of the light beams $b_{ + }$ and $b_{ - }$ is shown to take place by the two factors, the anisotropy of the one-way speed of light in inertial frames and the difference between the rotation radii of the segments $L_{1}$ and $L_{2}$. As the rotation radii are different their tangential speeds are different. The segments can be considered to belong to respective inertial frames during the travels of $b_{ + }$ and $b_{ - }$. As shown in (24), the one-way speed of light is anisotropic in inertial frames, which agrees with the experimental results of the generalized Sagnac effect.

Though inertial frames are not isotropic, regarding them as isotropic with the introduction of the standard synchronization, we can exactly obtain some physical quantities that are independent of synchronization schemes. These quantities can be accurately calculated using only relative velocities with no knowledge of absolute velocities. It is because the first rows of ${\mathbf{T}}_{L} ({\varvec{\beta}}_{j} ,\;{\varvec{\beta}}_{i} )$ and ${\mathbf{T}}_{L} ({\varvec{\beta}}_{ji} )$ are the same. As far as the experiments associated with circular motion are concerned, the Solar System or the Earth frame can be considered an inertial frame $S_{i}$ during a short time of test. Accordingly, we have obtained the exact time differences through the standard synchronization of *S*_i_ that is not isotropic.

### Supplementary Information

Supplementary Information.

## Data Availability

All data generated or analyzed during this study are included in this published article and its supplementary information file.
