# Supplementary material for: Relativistic analysis of the Michelson-Gale experimental result
Source: Sci Rep. 2024 Apr 30;14:9956. doi: 10.1038/s41598-024-60515-7 (PMC11061132; doi:10.1038/s41598-024-60515-7)
Supplement: Supplementary file 1 — Supplementary Information. [file 41598_2024_60515_MOESM1_ESM.pdf]

Derivation of (18)

From (6),

$$\beta'_m = r'_m \omega' / c = \gamma_m^2 \beta_m, \quad m = 1, 2\,. \tag{32}$$

The r'_2 is approximately given by

$$r'_2 = R' \sin(\alpha_1 - \Delta\alpha) \approx R'(\sin\alpha_1 - \Delta\alpha \cos\alpha_1)\,. \tag{33}$$

Using (32) and (33) and neglecting the terms with higher degrees than β_1 or β_2, we have

$$\xi_{21}\beta_2 l'_{w2} = \frac{r_2'^2 \omega' \Delta\widetilde{\phi}'}{c\gamma_1\gamma_2} \approx \frac{\omega' R'^2 \Delta\widetilde{\phi}'}{c}(\sin_z^2 \alpha_1 - 2\Delta\alpha \sin\alpha_1 \cos\alpha_1) \tag{34}$$

and

$$\beta_1 l'_{w1} - \xi_{21}\beta_2 l'_{w2} \approx \frac{2\omega' R'^2 \Delta\widetilde{\phi}' \Delta\alpha \sin\alpha_1 \cos\alpha_1}{c} = \frac{2\omega' l'_{w1} l'_h \cos\alpha_1}{c}\,. \tag{35}$$

Substituting (35) into (17) yields

$$\Delta t'_d \approx \frac{4\omega' l'_{w1} l'_h \cos\alpha_1}{c^2}\,. \tag{36}$$

Then the fringe shift, which is given by $N = c\Delta t'_d / \lambda$ in the first-order approximation, is expressed as (18).
